# Personalized Risk Assessment in Never, Light, and Heavy Smokers in a prospective cohort in Taiwan

**DOI:** 10.1038/srep36482

**Published:** 2016-11-02

**Authors:** Xifeng Wu, Chi Pang Wen, Yuanqing Ye, MinKwang Tsai, Christopher Wen, Jack A. Roth, Xia Pu, Wong-Ho Chow, Chad Huff, Sonia Cunningham, Maosheng Huang, Shuanbei Wu, Chwen Keng Tsao, Jian Gu, Scott M. Lippman

**Affiliations:** 1 Department of Epidemiology, and Cardiovascular Surgery, The University of Texas MD Anderson Cancer Center, Houston, TX, USA; 2 Institute of Population Health Science, National Health Research Institutes, Zhunan, Taiwan; 3 China Medical University Hospital, Taichung, Taiwan; 4 Department of Thoracic and Cardiovascular Surgery, The University of Texas MD Anderson Cancer Center, Houston, TX, USA; 5 Department of Radiological Sciences, University of California at Irvine, Irvine, CA, USA; 6 MJ Health Management Institution, Taipei, Taiwan; 7 UC San Diego Moores Cancer Center, San Diego, California, USA

## Abstract

The objective of this study was to develop markedly improved risk prediction models for lung cancer using a prospective cohort of 395,875 participants in Taiwan. Discriminatory accuracy was measured by generation of receiver operator curves and estimation of area under the curve (AUC). In multivariate Cox regression analysis, age, gender, smoking pack-years, family history of lung cancer, personal cancer history, BMI, lung function test, and serum biomarkers such as carcinoembryonic antigen (CEA), bilirubin, alpha fetoprotein (AFP), and c-reactive protein (CRP) were identified and included in an integrative risk prediction model. The AUC in overall population was 0.851 (95% CI = 0.840–0.862), with never smokers 0.806 (95% CI = 0.790–0.819), light smokers 0.847 (95% CI = 0.824–0.871), and heavy smokers 0.732 (95% CI = 0.708–0.752). By integrating risk factors such as family history of lung cancer, CEA and AFP for light smokers, and lung function test (Maximum Mid-Expiratory Flow, MMEF_25–75%_), AFP and CEA for never smokers, light and never smokers with cancer risks as high as those within heavy smokers could be identified. The risk model for heavy smokers can allow us to stratify heavy smokers into subgroups with distinct risks, which, if applied to low-dose computed tomography (LDCT) screening, may greatly reduce false positives.

Lung cancer is the leading contributor to cancer incidence and mortality worldwide^1^,^2^,^3^. The landmark National Lung Screening Trial (NLST) evaluated the benefits of low-dose computed tomography (LDCT) for screening of old-aged and heavy smokers (≥30 pack-years) and found that annual screening by LDCT yielded a relative reduction of lung cancer mortality of 20% among those screened when compared to chest radiography, with a caveat of potential harms from high false positives, over-diagnoses, economic burden and repeated radiation^4^. The current recommendation for lung cancer screening by LDCT focused mainly on those heavy smokers with at least 30 pack years. However, not all lung cancer comes from heavy smokers. In fact, it was estimated that only about a quarter of currently diagnosed lung cancer patients in the U.S. meet the strict NLST eligibility criteria (age 55–74, ≥30 pack-years smoking history)^5^. While focusing on heavy smokers is indeed a priority, a substantial portion of lung cancer cases continue to occur in light smokers and never smokers.

By design, light smokers and never smokers are not eligible for LDCT screening because they were assumed to have too low a risk for lung cancer. However, it is very likely that some of these individuals could have a risk of lung cancer similar to heavy smokers. To identify such individuals, additional risk factors working in tandem, other than smoking history, will be needed to create accurate risk models within light and never smokers. On the other hand, although LDCT could reduce mortality by 20%, the high false positive rate (96.4%) observed in the NLST calls for more accurate risk stratification among heavy smokers^4^. The UK Lung Screening (UKLS) Trial became the first trial to set up a threshold for pre-selecting screening population with an estimated risk of at least 5% of developing lung cancer in the next 5 years using the Liverpool Lung Project (LLP) risk model^6^. The American Association for Thoracic Surgery (AATS) guidelines call for annual lung cancer screening with LDCT for those starting at age 50 years with a 20 pack-year history if there is an additional cumulative risk of developing lung cancer of 5% or greater in the next 5 years^7^. Over the past decade, a concerted effort has been made to develop personalized risk prediction models for lung cancer^8^. Early reports yielded only modest discriminatory power with an area under the curve (AUC) of 0.72 or lower^9^,^10^,^11^. More recent models drawing on data collected by the Prostate, Lung, Colorectal, and Ovarian Cancer Screening Trial (PLCO) and the multi-center European Prospective Investigation into Cancer and Nutrition (EPIC) cohort which focused on smokers, have yielded improved discriminatory power with an AUC of 0.80–0.86 in the modeling population^12^,^13^,^14^. These existing models have primarily incorporated only limited demographic factors (e.g., age, gender, and smoking history) and recognized clinical risk variables (e.g., chronic obstructive pulmonary disease (COPD) and pneumonia).

In this study, based on analyzing clinical, biomarker and other (e.g., lung function tests) data from a large prospective cohort in Taiwan, we developed integrative lung cancer prediction models for heavy smokers, light smokers and never smokers for 5-year and 10-year probability.

## Results

### Characteristics of Cohort Participants

Among the 395,875 participants, there were a total of 1,117 incident lung cancer diagnoses. The mean ages were 40.4 for the whole cohort and 60.2 for the lung cancer cases. Categorization of the cohort by age group showed that the percentage of lung cancer cases increased from 0.07% in those of age <50 years to 1.95% in those of age ≥70 years. Over half (52%) of the cohort was female and 38% of the lung cancer cases occurred in females; translating to sex-specific incidences of 0.21% for females and 0.32% for males. Owing to the high percentage (71%) of never smokers in this cohort, 47% of the lung cancer cases occurred in never-smokers. Besides age, gender, and smoking, other variables associated with lung cancer included BMI, physical activity, and history of cancer (Table 1 and Supplemental Table 1).

### Cox Multivariate Analyses of Laboratory Test and Medical Examination Variables

We selected only those variables that were significant based on Cox multivariate regression analysis to develop risk prediction models for lung cancer (Supplemental Table 1). Significant risk factors among the laboratory test and medical examination variables included low MMEF (0–48 ml/sec versus >103 ml/sec, HR = 2.26, 95% CI = 1.84–2.78), and increasing levels of bilirubin (highest quartile versus lowest quartile, HR = 1.42, 95% CI = 1.19- 1.70), AFP (≥1.8 ng/ml versus <1.8 ng/ml, HR = 1.38, 95% CI = 1.12–1.70), CEA (>7.0 ng/ml versus <1.5 ng/ml, HR = 7.68, 95% CI = 6.07–9.73), and CRP (highest versus lowest quartile, HR = 1.55, 95% CI = 1.20–1.99). Serum bilirubin displayed a pattern of decreasing risk with increasing quartile levels, reaching a borderline significance when comparing the lowest to the highest quartile (HR = 1.16, 95% CI = 0.96 to 1.40). Additional analyses were performed in never smokers, light smokers (pack-years < 30) and heavy smokers (pack-years ≧30). The significant variables for never smokers include age, gender, BMI, family history of lung cancer, AFP, MMEF, and CEA, the significant variables for light smokers include age, sex, smoking status, smoking pack-year, family history of lung cancer, AFP, and CEA, and the significant variables for heavy smokers include age, sex, smoking status, smoking intensity, BMI, MMEF, and CEA (Table 2).

### Risk Modeling

**Figure 1:**
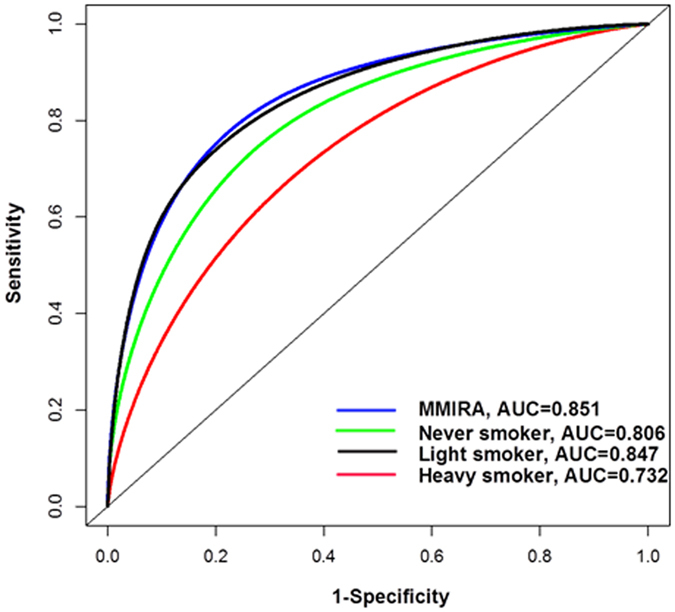
Discriminatory accuracy of lung cancer prediction models from MD Anderson – MJ Group Integrative Risk Assessment (MMIRA) in overall, heavy, light and never smokers for lung cancer risk. Discriminatory accuracy for predicting lung cancer risk within 10 years was assessed by receiver operator characteristics (ROC) analysis calculating area under the curve (AUC).

We then developed an integrative risk prediction model for the overall cohort, named the MD Anderson – MJ Group Integrative Risk Assessment (MMIRA) model based on variables in Table 2. We generated a time-dependent ROC curve, which yielded an AUC of 0.851 (95% CI = 0.840 to 0.862) (Fig. 1). We calculated the C-index using internal validation by splitting the overall dataset into equally sized training and validation sets (Supplemental Table 2). Concordance was excellent, with only minor attenuation when moving from the training set (0.854) to the validation set (0.848) for the overall. We were able to demonstrate good calibration agreement between the observed and predicted probability of no events within the 10-year time frame (Supplemental Fig. S1). We also generated separate models in never smokers, light smokers, and heavy smokers (Fig. 1). The AUC of 0.806 (95% CI = 0.790 to 0.819) and 0.847 (95% CI = 0.824 to 0.871) showed excellent predictive power in never-smokers and light smokers. Excellent concordance was also observed with minor attenuation from training to validation set for never smokers (0.795 to 0.822), light smokers (0.830 to 0.868), and heavy smokers (0.733 to 0.744) (Supplemental Table 2). In addition, we generated separate models in former smokers and current smokers with AUCs of 0.873 (95% CI = 0.829 to 0.879) and 0.875 (95% CI = 0.864 to 0.887), respectively (Supplemental Fig. S2). Further analysis showed that the positive predictive value for overall, never smokers, light smokers, heavy smokers, former smokers, and current smokers were 0.67%, 0.43%, 0.48%, 2.88%, 1.55%, and 1.73%, respectively (Supplementary Table 3).

### Application of risk prediction model

**Figure 2:**
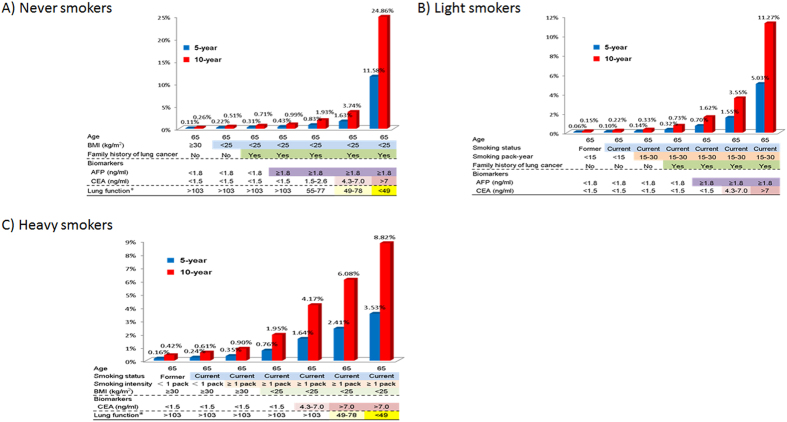
Application of the integrative risk prediction models in never smokers, light smokers, and heavy smokers (smoking pack-year ≥30) to predict absolute risk of developing lung cancer in 5 year and 10 years for hypothetical individuals, with different risk profiles by the addition of different risk factors.

We applied the MMIRA models developed in never smokers, light smokers, and heavy smokers to predict probability of developing lung cancer in 5 years and 10 years to hypothetical individuals of age 65 with a range of risk profiles (Fig. 2). For a 65-year old never smoker with relatively low risk profile (BMI ≥ 30, negative family history of lung cancer), the predicted risk of developing lung cancer was 0.11% in 5-years and 0.26% in 10-years. However, the predicted probability of developing lung cancer increased to the range of 0.22 to 11.58% in 5-years and the range of 0.51% to 24.86% in 10-years for the addition of one to five risk factors (BMI, positive family history of lung cancer, AFP, CEA, and MMEF). Similarly, for a 65-year old person who is light smoker, the probability of developing cancer increases from 0.06% to 5.03% in 5 years, and from 0.15% to 11.27% in 10 years. For a 65-year old person who is a heavy smoker, the probability of developing cancer increases from 0.16% to 3.53% in 5 years, and from 0.42% to 8.82% in 10 years.

**Figure 3:**
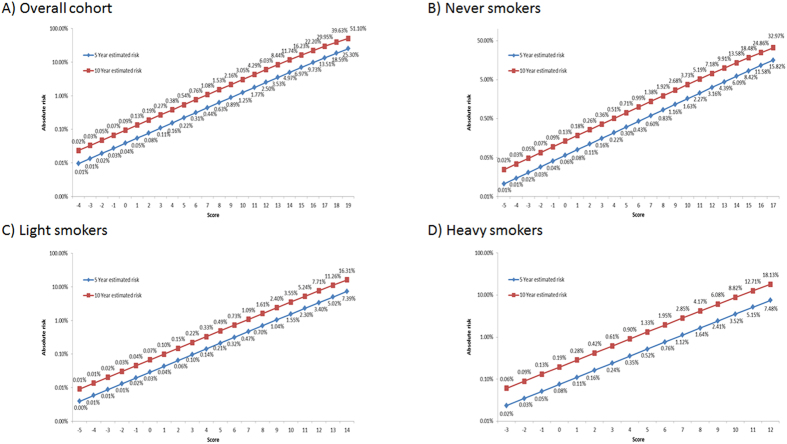
Predicted probability of lung cancer risk based on the risk scores from MMIRA models (**A**) in overall population, (**B**) in never smokers, (**C**) in light smokers, and (**D**) in heavy-smokers. Blue line – predicted probability in 5-year. Red line- predicted probability in 10 year.

We also assigned risk scores to each risk factor based on the strength of the association (Supplementary Table 4). The higher HR a risk factor conferred, the higher the risk score was assigned to the risk factor. For example, in the age category, age 50–59 was the reference group and the assigned score was 0, age <50 was protective with an assigned score of −4, whereas the assigned scores for age 60–69 and age ≥70 were 2 and 3, respectively. The risk scores for all cohort participants ranged from −4 to 19 for overall cohort: for never smokers, −5 to 17; for light smokers, −5 to 14; and for heavy smokers, −3 to 12. Figure 3 depicts the probability of developing lung cancer in 5 and 10 years as a function of increasing risk scores. For example, for never smokers with a score of 15, the corresponding risk would be 8.42% and 18.48% in 5 and 10 years, respectively (Fig. 3B). Similarly, we could use risk scores to stratify light smokers into 20 categories with the 5-year lung cancer probability ranging from 0.00% to 7.39% and stratify heavy smokers into 16 categories with the 5-year lung cancer probability ranging from 0.02% to 7.48% (Fig. 3C,D).

## Discussion

It was estimated that 26.7% of lung cancer cases occurred among heavy smokers who meet NLST eligibility criteria in the U.S.^5^. The growing desire to extend LDCT screening beyond heavy smokers is understandable, particularly among the overwhelming majority of Asian women who were inflicted with lung cancer but never smoked (70% to 90%). In this cohort, more than 70% of lung cancer occurred in female never smokers. These high lung cancers in Asian women came mainly from second hand smoke from their fathers, brothers, and spouses, living in a small enclosed space, thus the second hand smoking rate could reach 75% in their earlier years throughout their life. The LDCT screening results in heavy smokers has elevated the public expectation for targeted screening for high-risk groups other than heavy smokers. In this paper, we have developed robust risk prediction models for never and light smokers in Asia, in addition to more accurately identify higher risk subjects in heavy smokers. As new findings, other than the smoking information and family history, four clinically common biomarkers, CEA, AFP, CRP and bilirubin, as well as a specific lung function test, were found to be uniquely useful in identifying high risk individuals. These biomarkers and the lung function test divided never smokers, light smokers, and heavy smokers into distinct groups with a range of 5-year lung cancer probability. Never-smokers with risk scores of 14 and above (Fig. 3C) and light smokers with risk scores of 13 and above would have an absolute cancer risk above 5% in five years, a risk threshold level suggested by UKLS and AATS to start the LDCT screening^6^,^7^.

There have been attempts to use additional data to improve the discriminative performance of risk stratification and participant selection for LDCT screening, most notably the LLP model, which added 4 history questions (history of pneumonia, personal history of cancer, asbestos exposure, and family history of lung cancer) and has been applied to the UKLS trial. Our study added more risk factors including laboratory biomarkers and a lung function test and was able to identify those with cancer risk exceeding the 5% threshold in 5-year probability set up by the UKLS and AATS^6^,^7^. Our model incorporated several unique predictors of lung cancer risk, including MMEF as an index of airway obstruction, and the serum markers CEA, bilirubin, AFP, and CRP. These covariates have not been integrated into current lung cancer risk prediction models (Supplemental Table 5) because such data are often unavailable for population-based cohort studies.

Spirometry has been used to demonstrate airflow obstruction, and can also suggest restrictive ventilator impairment. In lung cancer risk screening, spirometry is particularly valuable among never smokers. Different parameters of lung function test have been suggested to evaluate their relationships with lung cancer risks, such as COPD, FEV_1_% or Forced Expiratory Flow in the middle half of FVC (MMEF_25–75%_). Incremental reduction of FEV_1_ values has been strongly associated with lung cancer risk, independent of smoking^15^. COPD, as defined by the GOLD [Global Initiative for Chronic Obstructive Lung Disease] criteria with FEV1/FVC < 0.7, was also known to be a strong risk factor for lung cancer, in both smokers and never smokers. Consistent with the literature, we found, in our cohort, either FEV1 or COPD a strong risk factor for lung cancer. However, in our final comparison among parameters, MMEF_25–75%_ turned out to be the most sensitive indicator for lung cancer risk after a multivariate analysis. Therefore, the risk score sheet relied on the values of MMEF_25–75%_ in our modeling. The most likely explanation for reduced lung function as a lung cancer risk is that it reflects airway inflammation, a prodromal phase for lung cancer risk. Airway inflammation could present itself as either obstructive or restrictive impairment, with the more impairment the higher the risk. Another possibility is that the reduced lung function may impair the ability to clear inhaled carcinogens from their airways, which could lead to increased contact time between carcinogens and airway epithelial cells. These mechanisms probably facilitated MMEF_25–75%_ as a lung cancer risk not only for smokers but also for never smokers, a feature important in our search for high risk individuals among never smokers. In our cohort, the lowest 8% MMEF_25–75%_ of overall subjects had doubled their cancer risks and contributed 22% of all lung cancer cases, resulting in a multivariate adjusted HR at 2.06.

The CEA glycoprotein is an established tumor marker for colorectal cancer, and has been evaluated as a prognostic or predictive marker for lung cancer^16^,^17^. In our cohort, high CEA, e.g. >7 ng/ml, showed marked increase in lung cancer risk, with adjusted HR 12.82 for never smokers and 4.21 for light smokers. This level of CEA, constituting 1% to 4% of the cohort subjects, served as an excellent screening biomarker in our prediction model. The AFP tumor marker is most commonly used to aid screening and diagnosis of liver cancer and monitor response to treatment^18^, but an increased level of AFP has also been associated with other malignancies. In this cohort, mild elevation of AFP, ≥1.8 ng/ml, was associated with 37% increase in lung cancer among never smokers.

Elevated level of serum CRP, a systemic marker of chronic inflammation, has been consistently associated increased risk of lung cancer^19^,^20^. With CRP greater than 10 mg/L, lung cancer risk increased by 54% in this cohort. For CRP at that level, there were 2% of the cohort and 7% of lung cancer cases.

We had reported the elevated lung cancer risk from low serum bilirubin, which has anti-oxidant properties. Others also reported that relatively low serum bilirubin was associated with higher risks of lung cancer and COPD in a cohort study^21^. This risk remained in our integrative prediction model for the overall group, but not subgroups we examined with multivariate analysis.

It is difficult to compare performance metrics between published risk prediction models for lung cancer as each have been developed in different populations with varying lengths of follow-up time. In an independent case-control study used to compare the early Bach, Spitz and LLP models, differences in model sensitivity and specificity were highlighted and only moderate discriminatory power (AUC = 0.66–0.69 for all models) was found^22^. The NLST trial defined high-risk criteria based solely on age and smoking history. It has been estimated that if the PLCO risk prediction model had been used to select individuals for LDCT screening in the NLST trial, 12 additional deaths attributable to lung cancer could have been prevented^14^. Using similar calculations, we estimate that an additional one death could have been avoided if updated PLCO_M2012_ model has been used, and an additional eight deaths due to lung cancer could have been avoided if our MMIRA model had been applied.

Our models for light smokers (AUC = 0.847) and never-smokers (AUC = 0.808) had excellent predictive power. By calculating a risk score based on risk factor profile, the 5-year lung cancer probability of a light smoker ranged from 0.00% to over 7.39%, and the probability of a never smoker ranged from 0.01% to over 15.82%. Never-smokers with risk scores of 14 and above and light smokers with risk scores of 13 and above would have an absolute cancer risk above 5% in five years, a threshold level suggested by UKLS and AATS to start the LDCT screening^6^,^7^. Thus, our prediction model is able to stratify light smokers and never smokers into subgroups with dramatically different probability of developing lung cancer, with a portion, albeit small, of them as high as those in heavy smokers. Clinicians and patients can consult our score sheet in making an informed decision for assessing the risks and benefits of screening with LDCT. As false positives and over-diagnosis had been a problem for using LDCT in heavy smokers, so will be the challenges for screening any group other than heavy smokers. However, individuals can make better-informed decisions based on his/her absolute risk of developing lung cancer.

Our study has a couple of limitations. First, although we have a relatively high level of discrimination, external validation of the models is required to determine predictive ability in an independent population. Nevertheless, internal calibration and bootstrap analysis of goodness of fit showed excellent agreement between predicted and observed events and between the two randomly selected subcohorts. Secondly, as the MJ model has not been validated in a non-Asian population, we do not know if it will function with same predictive power across other racial/ethnic groups.

In summary, using a very large prospective cohort of an Asian population, we have demonstrated the power of incorporating routine laboratory test data and medical evaluation variables into prediction algorithms for lung cancer. Our models should improve selection of high-risk individuals for targeted screening strategies. Additional studies are necessary to validate these results in independent cohorts and to extend the findings to other ethnic populations.

## Methods

### Study Population and Data Collection

All subjects were recruited by the MJ Health Group, Taiwan, to participate in a national health-screening program. The current analysis was conducted after a median 7.3 years (range = 0~11.9 years) of follow-up from 1996 to 2007. Details of the screening program have been reported previously^23^. In brief, each subject completed a comprehensive health history questionnaire to collect medical history and epidemiological data. Participants underwent hands-on physical examinations and submitted to a panel of 103 blood and medical tests including lung function tests. Informed consent was obtained from all participants. The study was approved by Institutional Review Boards at the National Health Research Institute in Taiwan and MD Anderson Cancer Center. All the methods of subject recruitment, data collection, and experiments were performed in accordance with relevant guidelines and regulations.

### Ascertainment of lung cancer

The national ID of each cohort participant was matched to the National Cancer Registry and National Death File in order to assess outcomes and events. As of 2008, the cohort had registered 1,117 new cases of lung cancer and 799 lung cancer deaths.

### Laboratory test and lung function test

Serum biomarkers CEA, bilirubin, AFP, and CRP were tested using the Abbott ARCHITECT ci8200. Airway obstruction was measured in a standard spirometry test and obstruction expressed as FEV1% or MMEF (maximum mid-expiratory flow).

### Statistical Analysis

Risk predictors were subjected to stepwise Cox proportional hazards regression analysis to identify significant predictors in multivariate models. Hazard ratios (HRs) and 95% confidence intervals (CIs) were estimated for each variable. Continuous variables, including MMEF, CEA, bilirubin, AFP, and CRP, were assessed by quartile or other cut-points as guided by cohort distribution. To evaluate the discriminatory accuracy of the risk prediction models, we assessed the goodness of fit by calculating the concordance index (C-index) and the area under the curve (AUC) from the receiver operating characteristic (ROC) curve analysis. We examined the goodness of fit for 10-year risk prediction in each of the training, validation, and full datasets. Bootstrap resampling was performed 100 times to generate the 95% CI for AUC. We created risk scores based on weighted sum of the identified risk factors in each model and the weights were based on the coefficients, βi, from the multivariate Cox regression model following the procedure by Sullivan *et al*. 2004^24^. For example, the increase in risk associated with a 5-year increase in age was first estimated as the constant B, followed by the calculation of risk score rounded to the nearest integer using the βi/B. All statistical tests were two-sided and only *P*-values less than 0.05 were considered statistically significant.

## Additional Information

**How to cite this article**: Wu, X. *et al*. Personalized Risk Assessment in Never, Light, and Heavy Smokers in a prospective cohort in Taiwan. *Sci. Rep.* **6**, 36482; doi: 10.1038/srep36482 (2016).

**Publisher’s note**: Springer Nature remains neutral with regard to jurisdictional claims in published maps and institutional affiliations.

## Supplementary Material

Supplementary Information

## Figures and Tables

**Table 1:** Cohort characteristics.

**Variables**	**Level**	**Overall**				**Never smoking**				**Light smoking**				**Heavy smokers**			
		N = 395,875		LC = 1,117		N = 281,111		LC = 525		N = 90,486		LC = 156		N = 2,4278		LC = 436	
		**N**	**%**	**n**	**%**	**N**	**%**	**n**	**%**	**N**	**%**	**n**	**%**	**N**	**%**	**n**	**%**
Age, mean(SD)		40(13.8)				40(13.8)				37(11.3)				57(10.1)			
Age	<50	293,040	74%	201	18%	207,061	74%	123	23%	79,619	88%	57	37%	6,360	26%	21	5%
	50–59	55,772	14%	260	23%	41,874	15%	146	28%	5,539	6%	26	17%	8,359	34%	88	20%
	60–69	34,531	9%	412	37%	23,988	9%	167	32%	3,673	4%	47	30%	6,870	28%	198	45%
	≥70	12,532	3%	244	22%	8,188	3%	89	17%	1,655	2%	26	17%	2,689	11%	129	30%
Sex	Male	191,120	48%	691	62%	92,763	33%	133	25%	75,272	83%	142	91%	23,085	95%	416	95%
	Female	204,755	52%	426	38%	188,348	67%	392	75%	15,214	17%	14	9%	1,193	5%	20	5%
Smoking status	Never	281,111	71%	525	47%												
	Former	23,750	6%	127	11%					18,167	20%	33	21%	5,583	23%	94	22%
	Current	89,867	23%	461	41%					71,406	80%	121	79%	18,461	77%	340	78%
Smoking intensity*	<1	86,089	75%	372	63%					76,304	84%	143	92%	9,785	40%	229	53%
(pack/per day)	≥1	28,675	25%	220	37%					14,182	16%	13	8%	14,493	60%	207	47%
Pack-year*	<15	66,516	58%	68	11%					66,516	74%	68	44%				
	15–29.9	23,970	21%	88	15%					23,970	26%	88	56%				
	≥30	24,278	21%	436	74%												
BMI (kg/m2)	<25	291,339	74%	781	70%	213,289	76%	362	69%	63,087	70%	101	65%	14,963	62%	318	73%
	25–29.9	89,563	23%	312	28%	57,869	21%	147	28%	23,471	26%	53	34%	8,223	34%	112	26%
	≥30	14,801	4%	24	2%	9,842	4%	16	3%	3,884	4%	2	1%	1,075	4%	6	1%
Physical activity	Inactive	206,056	52%	546	50%	145,219	52%	240	47%	47,992	53%	78	51%	12,845	53%	228	53%
	Low active	89,154	23%	177	16%	65,183	23%	89	17%	20,224	22%	24	16%	3,747	15%	64	15%
	Fully active	100,458	25%	374	34%	70,587	25%	186	36%	22,237	25%	50	33%	7,634	32%	138	32%
Family history of lung cancer	No	379,272	96%	1,059	95%	269,537	96%	497	95%	86,590	96%	146	94%	23,145	95%	416	95%
	Yes	16,603	4%	58	5%	11,574	4%	28	5%	3,896	4%	10	6%	1,133	5%	20	5%
Personal cancer history	No	390,895	99%	1,091	98%	277,098	99%	512	98%	89,911	99%	155	99%	23,886	98%	424	97%
	Yes	4,980	1%	26	2%	4,013	1%	13	2%	575	1%	1	1%	392	2%	12	3%
COPD	No	259,217	77%	532	51%	182,532	77%	284	58%	63,322	83%	84	60%	13,363	59%	164	39%
	Yes	20,748	6%	172	16%	13,354	6%	52	11%	4,592	6%	21	15%	2,802	12%	99	24%
	Restricted lung	55,009	16%	346	33%	39,993	17%	156	32%	8,662	11%	36	26%	6,354	28%	154	37%
Lung function																	
(1) FEV1 (%)	0–61	17,821	5%	143	13%	12,156	5%	42	8%	3,480	4%	15	10%	2,185	9%	86	20%
	62–73	26,408	7%	115	11%	19,037	8%	55	11%	5,041	6%	17	11%	2,330	10%	43	10%
	74–90	130,574	37%	328	30%	94,443	37%	158	31%	28,851	35%	44	29%	7,280	31%	126	30%
	91–105	126,604	35%	301	28%	87,987	35%	157	31%	31,343	38%	47	31%	7,274	31%	97	23%
	>106	55,741	16%	201	18%	38,656	15%	99	19%	12,951	16%	28	19%	4,134	18%	74	17%
(2) MMEF (ml/sec)	0–48	15,598	4%	174	16%	10,113	4%	63	12%	2,927	4%	19	13%	2,558	11%	92	22%
	49–54	11,722	3%	61	6%	8,014	3%	23	4%	2,543	3%	8	5%	1,165	5%	30	7%
	55–77	103,091	29%	317	29%	72,785	29%	141	27%	23,397	29%	34	22%	6,909	30%	142	33%
	78–103	147,469	41%	349	32%	105,481	42%	179	35%	34,297	42%	64	42%	7,691	33%	106	25%
	>103	79,651	22%	193	18%	56,163	22%	109	21%	18,568	23%	27	18%	4,920	21%	57	13%
Bilirubin (mg/dl)	M: ≤0.68; F ≤ 0.56	100,354	26%	310	28%	66,330	24%	117	23%	26,279	30%	47	31%	7,745	33%	146	34%
	M: 0.69–0.87; F: 0.57–0.70	95,562	25%	301	27%	67,021	24%	158	30%	22,087	25%	43	28%	6,454	27%	100	23%
	M: 0.88–1.11; F: 0.71–0.90	97,384	25%	286	26%	70,491	26%	123	24%	21,471	24%	41	27%	5,422	23%	122	28%
	M: ≥1.12; F: ≥ 0.91	93,105	24%	212	19%	70,402	26%	122	23%	18,536	21%	23	15%	4,167	18%	67	15%
AFP (ng/ml)	<1.8	82,806	22%	100	9%	64,516	24%	64	12%	16,862	19%	8	5%	1,428	6%	28	6%
	≥1.8	299,179	78%	1,000	91%	206,728	76%	450	88%	70,576	81%	147	95%	21,875	94%	403	94%
CEA (ng/ml)	<1.5	253,091	65%	315	28%	200,170	72%	226	43%	47,212	53%	33	21%	5,709	24%	56	13%
	1.5–2.5	86,595	22%	298	27%	53,648	19%	138	26%	25,533	29%	49	32%	7,414	31%	111	25%
	2.6–4.2	37,278	10%	266	24%	17,968	6%	93	18%	12,514	14%	44	28%	6,796	28%	129	30%
	4.3–7.0	10,588	3%	139	12%	4,060	1%	25	5%	3,317	4%	19	12%	3,211	13%	95	22%
	>7.0	2,757	1%	98	9%	993	0%	43	8%	687	1%	10	6%	1,077	4%	45	10%
CRP (mg/L)	0–1	259,145	71%	551	56%	187,169	72%	278	59%	59,607	72%	85	63%	12,369	56%	188	50%
	1.1–3	66,919	18%	227	23%	46,419	18%	113	24%	15,002	18%	28	21%	5,498	25%	86	23%
	3,1–10	30,639	8%	132	13%	20,681	8%	53	11%	6,693	8%	13	10%	3,265	15%	66	18%
	>10	8,499	2%	69	7%	5,537	2%	25	5%	1,900	2%	8	6%	1,062	5%	36	10%

LC = lung cancer. BMI = Body mass index. COPD = chronic obstructive pulmonary disease. FEV1 = forced expiratory volume in 1 second. MMEF = maximum midexpiratory flow. AFP = alpha-fetoprotein. CEA = carcinoembryonic antigen. CRP = C-reactive protein.

^*^Among smokers.

**Table 2:** Cox regression lung cancer prediction models from MD Anderson and MJ group Integrative Risk Assessment (MMIRA) in overall, never smokers, light smokers, and heavy smokers.

AUC		Overall	Never Smokers	Light Smokers	Heavy Smokers
		0.851	0.806	0.847	0.732
Variables		HR(95% CI)	HR(95% CI)	HR(95% CI)	HR(95% CI)
Age		1.07(1.07–1.08)	1.07(1.06–1.08)	1.08(1.07–1.10)	1.08(1.07–1.09)
Sex	Male	0.88(0.74–1.04)	0.71(0.58–0.87)	1.58(0.91–2.77)	1.11(0.71–1.74)
	Female	1.00	1.00	1.00	1.00
Smoking	Never	1.00			
	<30 pack-year	1.14(0.92–1.42)			
	≥30 pack-year	2.60(2.15–3.14)			
Smoking status	Former			1.00	1.00
	Current			1.64(1.08–2.48)	1.48(1.16–1.89)
Smoking pack year	<15			1.00	
	15~30			1.44(1.03–2.02)	
Smoking intensity	<1				1.00
(pack/per day)	≥1				1.46(1.20–1.79)
BMI	<25	2.11(1.35–3.30)	1.91(1.13–3.20)		2.48(1.11–5.58)
(kg/m2)	25–29.9	1.79(1.13–2.83)	1.55(0.91–2.64)		2.02(0.89–4.59)
	≥30	1.00	1.00		1.00
Family history of lung cancer	No	1.00	1.00	1.00	
	Yes	1.44(1.08–1.94)	1.66(1.13–2.45)	2.00(1.05–3.81)	
Personal cancer history	No	1.00			
	Yes	1.55(1.02–2.34)			
Bilirubin (mg/dl)	Male:≤0.68; Female:≤0.56	1.16(0.96–1.40)			
	Male:0.69–0.87; Female:0.57–0.70	1.06(0.88–1.29)			
	Male:0.88–1.11; Female:0.71–0.90	1.08(0.89–1.31)			
	Male:≥1.12; Female:≥0.91	1.00			
AFP (ng/ml)	<1.8	1.00	1.00	1.00	
	≥1.8	1.35(1.08–1.69)	1.37(1.05–1.80)	2.12(1.03–4.33)	
MMEF (ml/sec)	0–48	2.00(1.58–2.52)	1.87(1.36–2.57)		2.06(1.47–2.87)
	49–54	1.64(1.20–2.26)	1.46(0.93–2.29)		1.71(1.10–2.67)
	55–77	1.49(1.22–1.82)	1.24(0.96–1.60)		1.63(1.19–2.22)
	78–103	1.41(1.16–1.71)	1.19(0.93–1.51)		1.33(0.96–1.83)
	>103	1.00	1.00		1.00
CEA (ng/ml)	<1.5	1.00	1.00	1.00	1.00
	1.5–2.5	1.30(1.09–1.56)	1.24(0.99–1.55)	1.45(0.92–2.28)	1.15(0.83–1.59)
	2.6–4.2	1.64(1.35–1.99)	1.82(1.40–2.36)	1.88(1.18–3.01)	1.25(0.91–1.72)
	4.3–7.0	2.26(1.79–2.86)	1.76(1.14–2.71)	2.16(1.20–3.89)	1.79(1.27–2.52)
	>7.0	5.70(4.39–7.41)	12.82(9.10–18.07)	5.29(2.55–10.95)	2.55(1.70–3.83)
CRP (mg/L)	0–1	1.00			
	1.1–3	1.02(0.87–1.20)			
	3.1–10	0.96(0.78–1.17)			
	>10	1.38(1.07–1.78)			

BMI = Body mass index. AFP = alpha-fetoprotein. MMEF = maximum midexpiratory flow. CEA = carcinoembryonic antigen. CRP = C-reactive protein.
