# Author Correction: Personalized Risk Assessment in Never, Light, and Heavy Smokers in a prospective cohort in Taiwan

**DOI:** 10.1038/s41598-020-62322-2

**Published:** 2020-03-23

**Authors:** Xifeng Wu, Chi Pang Wen, Yuanqing Ye, MinKwang Tsai, Christopher Wen, Jack A. Roth, Xia Pu, Wong-Ho Chow, Chad Huff, Sonia Cunningham, Maosheng Huang, Shuanbei Wu, Chwen Keng Tsao, Jian Gu, Scott M. Lippman

**Affiliations:** 1 Department of Epidemiology, and Cardiovascular Surgery, The University of Texas MD Anderson Cancer Center, Houston, TX USA; 2 Institute of Population Health Science, National Health Research Institutes, Zhunan, Taiwan; 3 China Medical University Hospital, Taichung, Taiwan; 4 Department of Thoracic and Cardiovascular Surgery, The University of Texas MD Anderson Cancer Center, Houston, TX USA; 5 Department of Radiological Sciences, University of California at Irvine, Irvine, CA USA; 6 MJ Health Management Institution, Taipei, Taiwan; 7 UC San Diego Moores Cancer Center, San Diego, California USA

Correction to: *Scientific Reports* 10.1038/srep36482, published online 02 November 2016

This Article contains an error in the Introduction.

The text,

“On the other hand, although LDCT could reduce mortality by 20%, the high false positive rate (96.4%) observed in the NLST calls for more accurate risk stratification among heavy smokers.”

should read:

“On the other hand, although LDCT could reduce mortality by 20%, the high number of false positives within the positive results (96.4%) observed in the NLST calls for more accurate risk stratification among heavy smokers.”

