# Supplementary material for: Personalized Risk Assessment in Never, Light, and Heavy Smokers in a prospective cohort in Taiwan
Source: Sci Rep. 2016 Nov 2;6:36482. doi: 10.1038/srep36482 (PMC5090352; doi:10.1038/srep36482)
Supplement: Supplementary Information [file srep36482-s1.doc]

Personalized Risk Assessment in Never, Light, and Heavy Smokers in a prospective cohort in Taiwan

Xifeng Wu^1^*, Chi Pang [Wen^2,3^*](http://www.ncbi.nlm.nih.gov/pubmed?term=Wen CP%5BAuthor%5D&cauthor=true&cauthor_uid=23073549), Yuanqing Ye^1^, MinKwang [Tsai^2,3^](http://www.ncbi.nlm.nih.gov/pubmed?term=Tsai MK%5BAuthor%5D&cauthor=true&cauthor_uid=23073549), Christopher Wen^4^, Jack A. Roth^5^, Xia Pu^1^, Wong-Ho Chow^1^, Chad Huff^1^, Sonia Cunningham^1^, Maosheng Huang^1^, Shuanbei Wu^2,3^, Chwen Keng [Tsao^6^](http://www.ncbi.nlm.nih.gov/pubmed?term=Tsao CK%5BAuthor%5D&cauthor=true&cauthor_uid=23073549) , Jian Gu^1#^, Scott M. Lippman^7#^

^1^ Department of Epidemiology, and ^4^ Department of Thoracic and Cardiovascular Surgery, The University of Texas MD Anderson Cancer Center, Houston, TX, USA; ^2^ Institute of Population Health Science, National Health Research Institutes, Zhunan, Taiwan; ^3^ China Medical University Hospital, Taichung, Taiwan; ^4^Department of Radiological Sciences, University of California at Irvine, Irvine, CA; ^6^ MJ Health Management Institution, Taipei, Taiwan; ^7^UC San Diego Moores Cancer Center, San Diego, California.

*****Equal contribution as first authors

#Equal contribution as last authors

Correspondence to: Xifeng Wu, MD, PhD, Department of Epidemiology, The University of Texas MD Anderson Cancer Center, Houston, TX, USA, 1155 Pressler Street, Unit1340, Houston, Texas 77030; Tel: 713-745-2485; FAX: 713-792-2145; email: [xwu@mdanderson.org](mailto:xwu@mdanderson.org)

Supplemental Information

**Table S1. Age and sex adjusted main effects of risk factors with lung** cancer

|  |  | **Overall** | | **Never smokers** | | **Light smokers** | | **Heavy smokers** | |
| --- | --- | --- | --- | --- | --- | --- | --- | --- | --- |
|  |  | **HR(95 % CI)** | **NNS** | **HR(95 % CI)** | **NNS** | **HR(95 % CI)** | **NNS** | **HR(95 % CI)** | **NNS** |
| Age, mean(SD) |  | 1.09(1.08-1.09) |  | 1.08(1.07-1.09) |  | 1.09(1.08-1.10) |  | 1.08(1.07-1.09) |  |
| Age | <50 | 0.15(0.12-0.18) | 1,458 | 0.18(0.14-0.23) | 1,683 | 0.14(0.09-0.22) | 1,397 | 0.38(0.23-0.60) | 303 |
|  | 50-59 | 1.00 | 215 | 1.00 | 287 | 1.00 | 213 | 1.00 | 95 |
|  | 60-69 | 2.30(1.97-2.69) | 84 | 1.91(1.53-2.38) | 144 | 2.14(1.32-3.45) | 78 | 2.72(2.11-3.49) | 35 |
|  | ≥ 70 | 3.83(3.22-4.56) | 51 | 3.17(2.44-4.13) | 92 | 2.72(1.58-4.68) | 64 | 4.80(3.66-6.30) | 21 |
| Sex | Male | 1.66(1.47-1.87) | 277 | 0.79(0.65-0.96) | 697 | 1.79(1.03-3.10) | 530 | 1.13(0.72-1.77) | 55 |
|  | Female | 1.00 | 481 | 1.00 | 480 | 1.00 | 1,087 | 1.00 | 60 |
| Smoking status | Never | 1.00 | 535 |  |  |  |  |  |  |
|  | Former | 1.55(1.25-1.92) | 187 |  |  | 1.00 | 551 | 1.00 | 59 |
|  | Current | 2.80(2.40-3.27) | 195 |  |  | 1.97(1.33-2.92) | 590 | 1.69(1.33-2.13) | 54 |
| Smoking intensity* | <1 | 1.00 | 231 |  |  | 1.00 | 534 | 1.00 | 43 |
| (pack/per day) | ≥ 1 | 1.93(1.63-2.28) | 130 |  |  | 1.20(0.67-2.17) | 1,091 | 1.43(1.18-1.74) | 70 |
| Pack-year* | <15 | 1.00 | 978 |  |  | 1.00 | 978 |  |  |
|  | 15-29.9 | 1.77(1.29-2.44) | 272 |  |  | 1.71(1.24-2.37) | 272 |  |  |
|  | ≥ 30 | 3.45(2.62-4.54) | 56 |  |  |  |  |  |  |
| BMI | <25 | 2.05(1.36-3.08) | 373 | 1.74(1.05-2.87) | 589 | 2.57(0.63-10.42) | 625 | 2.72(1.21-6.10) | 47 |
| (kg/m2) | 25-29.9 | 1.69(1.11-2.56) | 287 | 1.47(0.88-2.46) | 394 | 2.91(0.71-11.97) | 443 | 2.01(0.89-4.58) | 73 |
|  | ≥ 30 | 1.00 | 617 | 1.00 | 615 | 1.00 | 1,942 | 1.00 | 179 |
| Physical activity | Inactive | 1.00 | 377 | 1.00 | 605 | 1.00 | 615 | 1.00 | 56 |
|  | Low active | 0.74(0.62-0.88) | 504 | 0.82(0.64-1.04) | 732 | 0.63(0.40-1.00) | 843 | 0.87(0.66-1.15) | 59 |
|  | Fully active | 0.77(0.67-0.88) | 269 | 0.96(0.79-1.16) | 380 | 0.72(0.50-1.04) | 445 | 0.82(0.66-1.01) | 55 |
| Family history of lung cancer | No | 1.00 | 358 | 1.00 | 542 | 1.00 | 593 | 1.00 | 56 |
|  | Yes | 1.58(1.21-2.06) | 286 | 1.65(1.12-2.41) | 413 | 1.81(0.95-3.43) | 390 | 1.30(0.83-2.04) | 57 |
| Personal cancer history | No | 1.00 | 358 | 1.00 | 541 | 1.00 | 580 | 1.00 | 56 |
|  | Yes | 1.36(0.92-2.02) | 192 | 1.08(0.62-1.88) | 309 | 0.62(0.09-4.43) | 575 | 2.17(1.22-3.87) | 33 |
| COPD | No | 1.00 | 487 | 1.00 | 643 | 1.00 | 754 | 1.00 | 81 |
|  | Yes | 1.36(1.14-1.63) | 121 | 1.00(0.74-1.35) | 257 | 1.13(0.69-1.85) | 219 | 1.57(1.21-2.03) | 28 |
|  | Restricted lung | 1.33(1.16-1.53) | 159 | 1.12(0.91-1.37) | 256 | 1.23(0.82-1.85) | 241 | 1.43(1.14-1.78) | 41 |
| FEV1 | 0-61 | 1.85(1.49-2.29) | 125 | 1.22(0.85-1.76) | 289 | 1.40(0.75-2.63) | 232 | 1.94(1.42-2.64) | 25 |
| (%) | 62-73 | 1.52(1.21-1.91) | 230 | 1.44(1.03-2.00) | 346 | 1.72(0.94-3.15) | 297 | 1.14(0.78-1.65) | 54 |
|  | 74-90 | 1.47(1.23-1.76) | 398 | 1.30(1.00-1.68) | 598 | 1.36(0.84-2.20) | 656 | 1.37(1.02-1.83) | 58 |
|  | 91-105 | 1.27(1.06-1.52) | 421 | 1.27(0.98-1.64) | 560 | 1.26(0.78-2.03) | 667 | 1.08(0.79-1.47) | 75 |
|  | >106 | 1.00 | 277 | 1.00 | 390 | 1.00 | 463 | 1.00 | 56 |
| MMEF | 0-48 | 2.26(1.84-2.78) | 90 | 1.96(1.43-2.68) | 161 | 1.87(1.03-3.41) | 154 | 2.18(1.56-3.04) | 28 |
| (ml/sec) | 49-54 | 1.84(1.38-2.45) | 192 | 1.48(0.94-2.31) | 348 | 1.71(0.77-3.77) | 318 | 1.89(1.21-2.95) | 39 |
|  | 55-77 | 1.51(1.26-1.80) | 325 | 1.26(0.98-1.62) | 516 | 1.14(0.69-1.90) | 688 | 1.74(1.28-2.37) | 49 |
|  | 78-103 | 1.33(1.11-1.58) | 423 | 1.16(0.92-1.48) | 589 | 1.70(1.08-2.66) | 536 | 1.34(0.97-1.85) | 73 |
|  | >103 | 1.00 | 413 | 1.00 | 515 | 1.00 | 688 | 1.00 | 86 |
| Bilirubin | M: ≤ 0.68; F ≤ 0.56 | 1.42(1.19-1.70) | 324 | 1.06(0.82-1.37) | 567 | 1.76(1.07-2.91) | 559 | 1.44(1.08-1.92) | 53 |
| (mg/dl) | M: 0.69-0.87; F: 0.57-0.70 | 1.26(1.06-1.50) | 317 | 1.23(0.97-1.56) | 424 | 1.55(0.93-2.57) | 514 | 1.05(0.77-1.44) | 65 |
|  | M: 0.88-1.11; F: 0.71-0.90 | 1.18(0.99-1.41) | 341 | 0.93(0.72-1.20) | 573 | 1.44(0.86-2.40) | 524 | 1.43(1.06-1.93) | 44 |
|  | M: ≥ 1.12; F: ≥ 0.91 | 1.00 | 439 | 1.00 | 577 | 1.00 | 806 | 1.00 | 62 |
| AFP | <1.8 | 1.00 | 828 | 1.00 | 1,008 | 1.00 | 2,108 | 1.00 | 51 |
| (ng/ml) | ≥ 1.8 | 1.38(1.12-1.70) | 299 | 1.31(1.01-1.71) | 459 | 2.34(1.15-4.79) | 480 | 1.06(0.73-1.56) | 54 |
| CEA | <1.5 | 1.00 | 803 | 1.00 | 886 | 1.00 | 1,431 | 1.00 | 102 |
| (ng/ml) | 1.5-2.5 | 1.40(1.19-1.65) | 291 | 1.29(1.03-1.60) | 389 | 1.65(1.05-2.58) | 521 | 1.25(0.91-1.73) | 67 |
|  | 2.6-4.2 | 2.01(1.69-2.39) | 140 | 1.88(1.46-2.43) | 193 | 2.21(1.39-3.51) | 284 | 1.45(1.06-1.99) | 53 |
|  | 4.3-7.0 | 2.95(2.39-3.64) | 76 | 1.79(1.18-2.74) | 162 | 2.61(1.46-4.67) | 175 | 2.27(1.63-3.17) | 34 |
|  | >7.0 | 7.68(6.07-9.73) | 28 | 12.69(9.04-17.80) | 23 | 6.19(3.01-12.76) | 69 | 3.32(2.24-4.92) | 24 |
| CRP | 0-1 | 1.00 | 470 | 1.00 | 673 | 1.00 | 701 | 1.00 | 66 |
| (mg/L) | 1.1-3 | 1.05(0.90-1.23) | 295 | 1.06(0.85-1.32) | 411 | 0.97(0.63-1.49) | 536 | 1.00(0.78-1.30) | 64 |
|  | 3,1-10 | 1.05(0.86-1.27) | 232 | 0.88(0.65-1.18) | 390 | 0.81(0.45-1.46) | 515 | 1.15(0.87-1.52) | 49 |
|  | >10 | 1.55(1.20-1.99) | 123 | 1.36(0.90-2.06) | 221 | 1.35(0.65-2.80) | 238 | 1.47(1.03-2.11) | 30 |

*among smokers

NNS=Number of subjects needed to screen to find one cancer, HR and 95% CI adjusted for age and gender when appropriated. *Smoking intensity and pack-year among ever smokers. LC=lung cancer. BMI=Body mass index. AFP=alpha-fetoprotein. COPD=chronic obstructive pulmonary disease. FEV1=forced expiratory volume in 1 second. MMEF=maximum midexpiratory flow. CEA=carcinoembryonic antigen. CRP=C-reactive protein.

**Table S2. The goodness of fit by C-index in lung cancer prediction models from MD Anderson and MJ group Integrative Risk Assessment (MMIRA) in overall, never smokers, light smokers, and heavy smokers**

|  | Overall | Never smokers |  | Light smokers | Heavy smokers |
| --- | --- | --- | --- | --- | --- |
| Training set | 0.854(0.837-0.865) | 0.822(0.799-0.844) |  | 0.868(0.822-0.897) | 0.733(0.704-0.763) |
| Validation set | 0.848(0.834-0.864) | 0.795(0.776-0.817) |  | 0.830(0.787-0.868) | 0.744(0.717-0.775) |
| Full dataset | 0.849(0.839-0.861) | 0.810(0.794-0.822) |  | 0.849(0.823-0.872) | 0.737(0.711-0.756) |

Table S3. Lung cancer classification accuracy for lung cancer prediction models

| **Criteria** | **Lung Cancer** | **(%)** | **No lung Cancer** | **(%)** | **Total** | **Predictive value** |
| --- | --- | --- | --- | --- | --- | --- |
| Overall Model |  |  |  |  |  |  |
| Criteria positive | 706 | 0.67 | 104648 | 99.33 | 105354 | PPV, 0.67% |
| Criteria negative | 237 | 0.11 | 216765 | 99.89 | 217002 | NPV, 99.89% |
| Never Smoking Model |  |  |  |  |  |  |
| Criteria positive | 179 | 0.43 | 41112 | 99.57 | 41291 | PPV, 0.43% |
| Criteria negative | 148 | 0.15 | 101243 | 99.85 | 101391 | NPV, 99.85% |
| Light Smoking Model |  |  |  |  |  |  |
| Criteria positive | 123 | 0.48 | 25403 | 99.52 | 25526 | PPV, 0.48% |
| Criteria negative | 29 | 0.05 | 60238 | 99.95 | 60267 | NPV, 99.95% |
| Heavy Smoking Model |  |  |  |  |  |  |
| Criteria positive | 189 | 2.88 | 6377 | 97.12 | 6566 | PPV, 2.88% |
| Criteria negative | 236 | 1.44 | 16168 | 98.56 | 16404 | NPV, 98.56% |
| Former Smoking Model |  |  |  |  |  |  |
| Criteria positive | 83 | 1.55 | 5255 | 98.45 | 5338 | PPV, 1.55% |
| Criteria negative | 22 | 0.15 | 14250 | 99.85 | 14272 | NPV, 99.85% |
| Current Smoking Model |  |  |  |  |  |  |
| Criteria positive | 409 | 1.73 | 23214 | 98.27 | 23623 | PPV, 1.73% |
| Criteria negative | 41 | 0.07 | 57017 | 99.93 | 57058 | NPV, 99.93% |

NPV negative predictive value, PPV positive predictive value

Positivity was defined as the 33th percentile of risk with cut-off as 2.54%, 1.44%, 0.14%, 13.43%, 1.25% for overall, never smoker, light smoker, heavy smoker, former smoker, and current smoker, respectively.

**Table S4. Assignment of risk scores in lung cancer prediction models from MD Anderson and MJ group Integrative Risk Assessment (MMIRA) in overall, never smokers, light smokers, and heavy smokers**

|  |  | Overall | Never Smokers | Light Smokers | Heavy smokers |
| --- | --- | --- | --- | --- | --- |
|  |  | Score | Score | score | Score |
|  |  | (-4~19) | (-5~17) | (-5~14) | (-3~12) |
| Age | <50 | -4 | -4 | -5 | -3 |
|  | 50-59 | 0 | 0 | 0 | 0 |
|  | 60-69 | 2 | 2 | 2 | 2 |
|  | ≥ 70 | 3 | 3 | 2 | 4 |
| Sex | Male | 0 | -1 | 1 | 0 |
|  | Female | 0 | 0 | 0 | 0 |
| Smoking | Never | 0 |  |  |  |
|  | <30 pack-year | 0 |  |  |  |
|  | ≥30 pack-year | 3 |  |  |  |
| Smoking | Former |  |  | 0 | 0 |
|  | Current |  |  | 1 | 1 |
| Pack year | <15 |  |  | 0 |  |
|  | 15~30 |  |  | 1 |  |
| Smoking Intensity | <1 |  |  |  | 0 |
|  | ≥ 1 |  |  |  | 1 |
| BMI | <25 | 2 | 2 |  | 2 |
| (kg/m^2^) | 25-29.9 | 2 | 1 |  | 2 |
|  | ≥ 30 | 0 | 0 |  | 0 |
| Family history of lung cancer | No | 0 | 0 | 0 |  |
|  | Yes | 1 | 1 | 2 |  |
| Personal cancer history | No | 0 |  |  |  |
|  | Yes | 1 |  |  |  |
| Bilirubin | Male: ≤ 0.68; Female: ≤ 0.56 | 0 |  |  |  |
| (mg/dl) | Male: 0.69-0.87; Female: 0.57-0.70 | 0 |  |  |  |
|  | Male: 0.88-1.11; Female: 0.71-0.90 | 0 |  |  |  |
|  | Male: ≥ 1.12; Female: ≥ 0.91 | 0 |  |  |  |
| AFP | <1.8 | 0 | 0 | 0 |  |
| (ng/ml) | >=1.8 | 1 | 1 | 2 |  |
| MMEF | 0-48 | 2 | 2 |  | 2 |
| (ml/sec) | 49-54 | 1 | 1 |  | 1 |
|  | 55-77 | 1 | 1 |  | 1 |
|  | 78-103 | 1 | 0 |  | 1 |
|  | >103 | 0 | 0 |  | 0 |
| CEA | <1.5 | 0 | 0 | 0 | 0 |
| (ng/ml) | 1.5-2.5 | 1 | 1 | 1 | 0 |
|  | 2.6-4.2 | 2 | 2 | 2 | 1 |
|  | 4.3-7.0 | 3 | 3 | 2 | 2 |
|  | >7.0 | 5 | 8 | 5 | 2 |
| CRP | 0-1 | 0 |  |  |  |
| (mg/L) | 1.1-3 | 0 |  |  |  |
|  | 3.1-10 | 0 |  |  |  |
|  | >10 | 1 |  |  |  |

BMI=Body mass index. MMEF=maximum midexpiratory flow. AFP=alpha-fetoprotein. CEA=carcinoembryonic antigen. CRP=C-reactive protein

**Table S5. Review of existing risk prediction models**

| **Model Name** | **Bach** | **SPITZ** | | | **SPITZ_expand** | | **LLP** | **Etzel_AA** | **PLCO2011** | | **EPIC** | | **PLCOM2012** | **Park** | **PLCO_all2014** | **LLPi** |
| --- | --- | --- | --- | --- | --- | --- | --- | --- | --- | --- | --- | --- | --- | --- | --- | --- |
| **Publication** | Bach et al. JNCI 2003 | Spitz et al. JNCI 2007 | | | Spitz et al. Cancer Prev Res 2008 | | Cassidy et al. Br J Cancer. 2008 | Etzel et al. Cancer Prev Res 2008 | Tammemagi et al. JNCI. 2011 | | Hoggart et at. Cancer Prev Res 2012 | | Tammemagi et al. NEJM 2013 | Park et al. PLoS One. 2013 | Tammemagi et al. Plos Med 2014 | Marcus et al. Cancer Prev Res 2015 |
| **Population** | All | Never smoker | Former smoker | Current smoker | Former smoker | Current smoker | All | African American, All | All | Ever smoker | Former smoker | Current smoker | Ever smoker | All | All | All |
| **Age** | √ |  |  |  |  |  |  |  | √ | √ |  |  | √ | √ | √ | √ |
| **Sex** | √ |  |  |  |  |  |  |  |  |  |  |  |  |  |  | √ |
| **BMI** |  |  |  |  |  |  |  |  | √ | √ |  |  | √ | √ | √ |  |
| **Race** |  |  |  |  |  |  |  |  |  |  |  |  | √ |  | √ |  |
| **Education** |  |  |  |  |  |  |  |  | √ | √ |  |  | √ |  | √ |  |
| **Personal cancer history** |  |  |  |  |  |  | √ |  |  |  |  |  | √ |  | √ | √ |
| **FH of Cancer** |  | √ | √ |  | √ |  |  |  |  |  |  |  |  |  |  |  |
| **FH of smoking related cancer** |  |  |  | √ |  | √ |  |  |  |  |  |  |  |  |  |  |
| **FH of LC** |  |  |  |  |  |  | √ |  | √ | √ |  |  | √ |  | √ | √ |
| **Cigarettes/per day** | √ |  |  |  |  |  |  |  |  |  | √ | √ | √ | √ | √ |  |
| **Smoking duration** | √ |  |  |  |  |  | √ |  | √ | √ | √ |  | √ |  | √ | √ |
| **Smoking status** |  |  |  |  |  |  |  | √ | √ | √ |  |  | √ | √ | √ |  |
| **Pack-years** |  |  |  | √ |  | √ |  | √ | √ | √ |  |  |  |  |  |  |
| **Cessation duration** | √ |  |  |  |  |  |  | √ |  | √ |  |  | √ |  | √ |  |
| **Chest X-ray** |  |  |  |  |  |  |  |  | √ | √ |  |  |  |  |  |  |
| **Age started smoking** |  |  |  |  |  |  |  |  |  |  | √ | √ |  | √ |  |  |
| **Age stopped smoking** |  |  | √ |  | √ |  |  | √ |  |  |  |  |  |  |  |  |
| **Asbestos exposure** | √ |  |  | √ |  | √ | √ | √ |  |  |  |  |  |  |  |  |
| **Wood dust exposure** |  |  | √ | √ | √ | √ |  | √ |  |  |  |  |  |  |  |  |
| **COPD** |  |  | √ | √ | √ | √ |  | √ | √ | √ |  |  | √ |  | √ | √ |
| **ETS** |  | √ |  |  |  |  |  |  |  |  |  |  |  |  |  |  |
| **Hay fever** |  |  | √ | √ | √ | √ |  | √ |  |  |  |  |  |  |  |  |
| **Pneumonia** |  |  |  |  |  |  | √ |  |  |  |  |  |  |  |  |  |
| **DNA repair capacity** |  |  |  |  | √ | √ |  |  |  |  |  |  |  |  |  |  |
| **Bleomycin sensitivity** |  |  |  |  | √ | √ |  |  |  |  |  |  |  |  |  |  |
| **Physical activity** |  |  |  |  |  |  |  |  |  |  |  |  |  | √ |  |  |
| **Fasting glucose level** |  |  |  |  |  |  |  |  |  |  |  |  |  | √ |  |  |
| **AUC (95% CI)** | AUC not reported, concor-  dance index: 0.72  . | 0.57 | 0.63 | 0.58 | 0.70 (0.66–0.74) | 0.73  (0.69–0.77) | 0.7 | Training:  0.75 (0.67–0.82); external validation:  0.63 (0.57–0.69) | Training00.857 external validation0.841 | Training: 0.805; external validation0.784 | 1-year: 0.830  (0.762-  0.899); 5-yrs: 0.715  (0.532-  0.898) | 1-yr: 0.824  (0.783-  0.865); 5-yrs: 0.767  (0.701-  0.832) | Training:  0.803  external validation  0.797 | C-  statistics  0.871  (0.867-  0.876) | Training:  0.859  external validation:  0.848  Never smoker only  0.662 | C-statistics: 0.849  (0.829-0.873) |

**Figure S1. Internal calibration of prediction. Integrative Model for lung cancer risk based on the extent of agreement between the predicted and observed probabilities of no events in 10 years. A calibration plot was generated for the overall cohort data. The *dashed line*s indicate the reference line for the ideal model. *Solid dots* mark the apparent predicted value for risk subgroups, while *cross symbols* mark the bootstrapping bias-corrected predictions. *Vertical bars* indicate 95% confidence intervals around the apparent prediction.**

**Figure S2. Receiver operating characteristic (ROC) curve and area under curve (AUC) of lung cancer prediction models from MD Anderson – MJ Group Integrative Risk Assessment (MMIRA) in current and former smokers.**

**
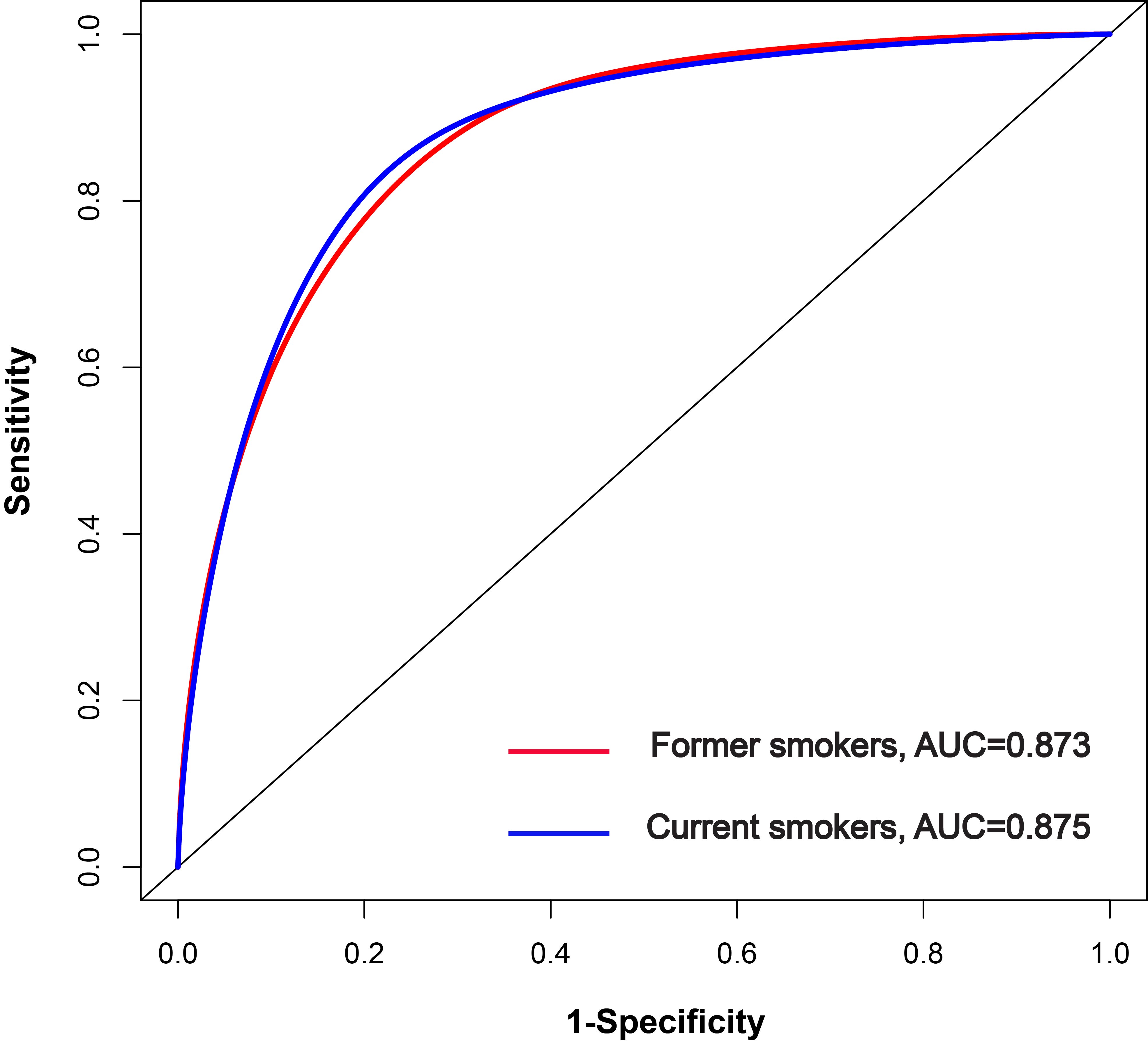
**
